# Supplementary material for: Integration of Gold Nanoparticles into Crosslinker-Free Polymer Particles and Their Colloidal Catalytic Property
Source: Nanomaterials (Basel). 2023 Jan 19;13(3):416. doi: 10.3390/nano13030416 (PMC9920725; doi:10.3390/nano13030416)
Supplement: Supplementary file 1 [file nanomaterials-13-00416-s001.zip › nanomaterials-2125910-supplementary.pdf]

Integration of Gold Nanoparticles into Crosslinker-Free Polymer Particles and Their Colloidal Catalytic Property

Jian Hou[1], Bin Li,[1,2,*], Wongi Jang,[3] Jaehan Yun,[3] Faith M. Eyimegwu,[3] and Jun-Hyun Kim[3,*]

[1] School of Intelligent Manufacturing, Luoyang Institute of Science and Technology, Luoyang 471023, China
[2] Henan International Joint Laboratory of Cutting Tools and Precision Machining, Luoyang Institute of Science and Technology, Luoyang 471023, China
[3] Department of Chemistry, Illinois State University, Normal, IL 61790-4160, USA
* Correspondence: libin@lit.edu.cn (B.L.) and jkim5@ilstu.edu (J.-H.K.)

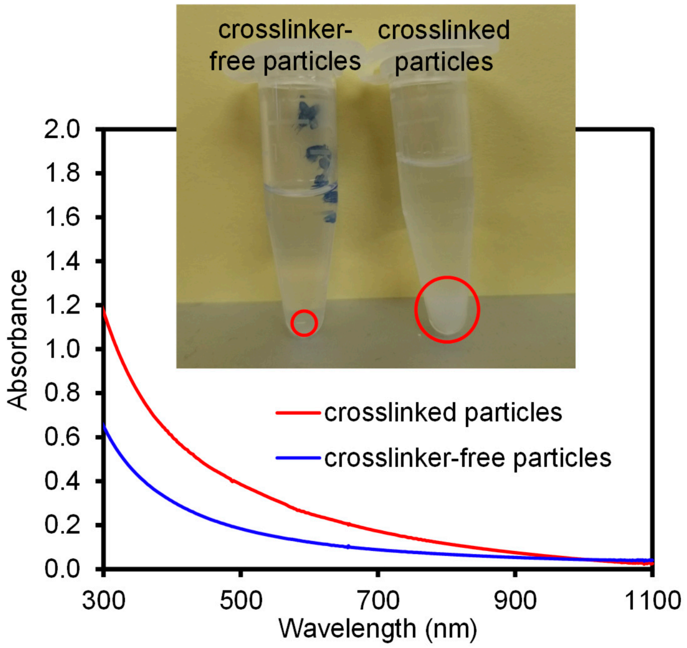

Figure S1. A digital photo and absorbance of crosslinker-free and crosslinked PNIPAM particles in water after centrifugation (red circles indicate the precipitated particles).

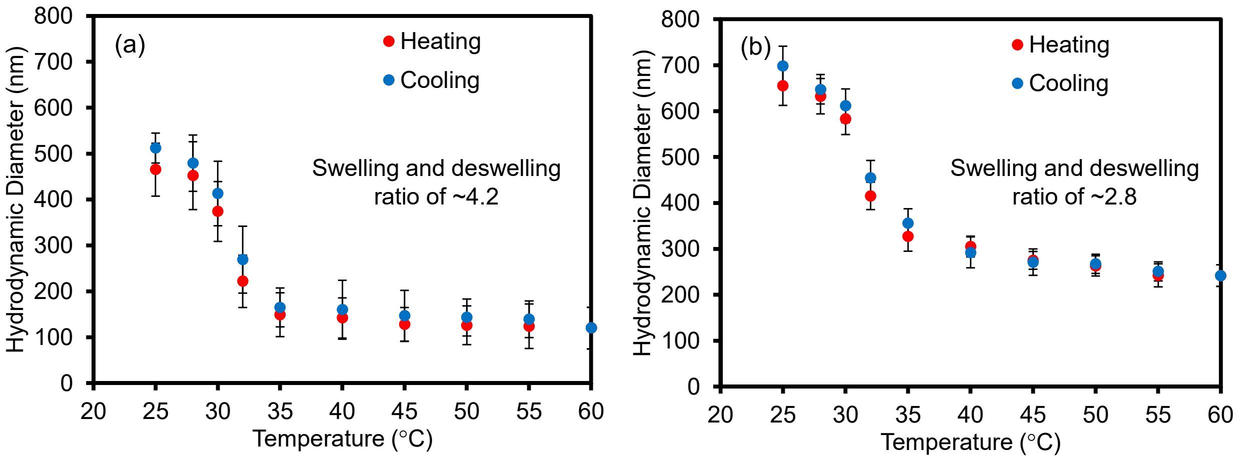

Figure S2. Hydrodynamic diameter changes of (a) crosslinker-free and (b) crosslinked PNIPAM particles as a function of temperature.

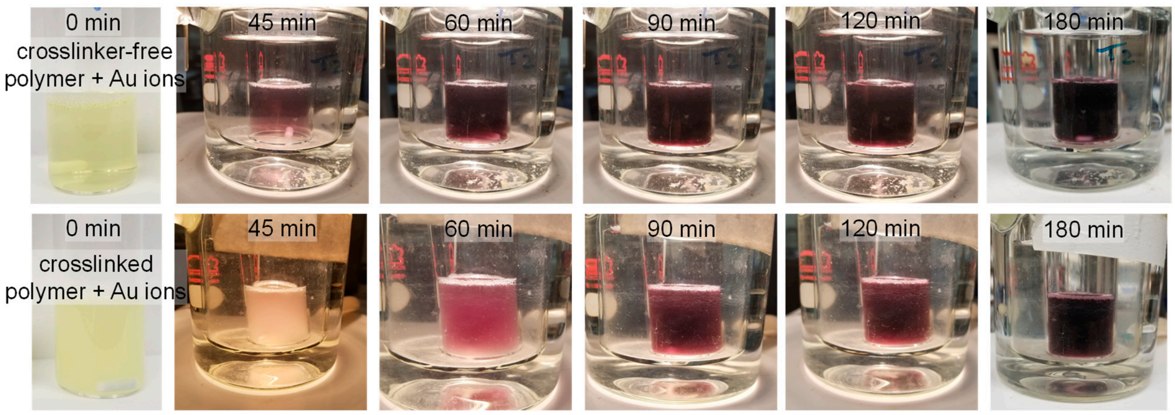

Figure S3. The reduction process of gold ions in the presence of crosslinker-free (top) and crosslinked (bottom) polymer particles under a solar-simulated light source as a function of time.

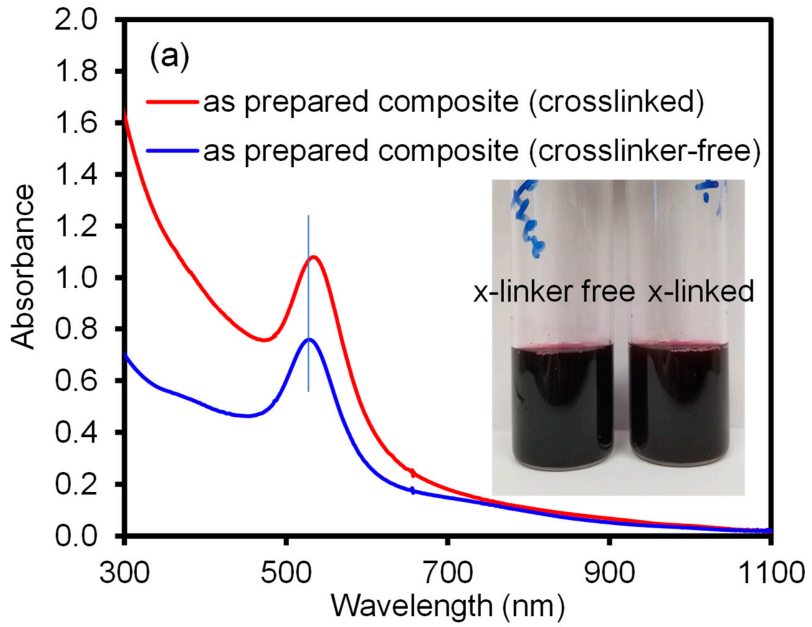

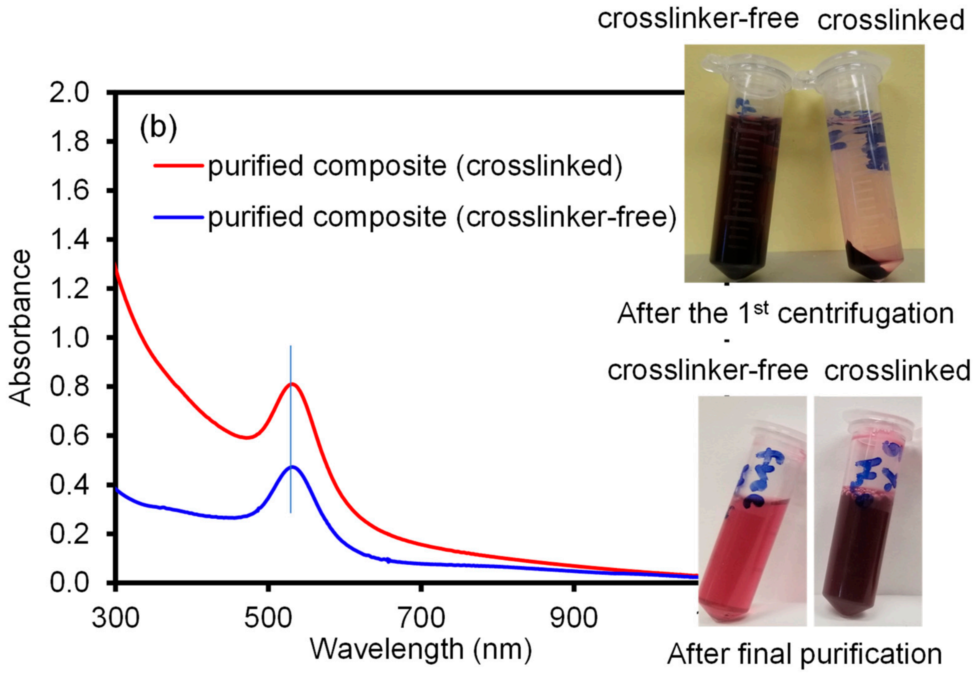

Figure S4. Absorption (i.e., SPR) patterns of crosslinker-free and crosslinked composite particles (a) before and (b) after the purification as well as their corresponding photos.

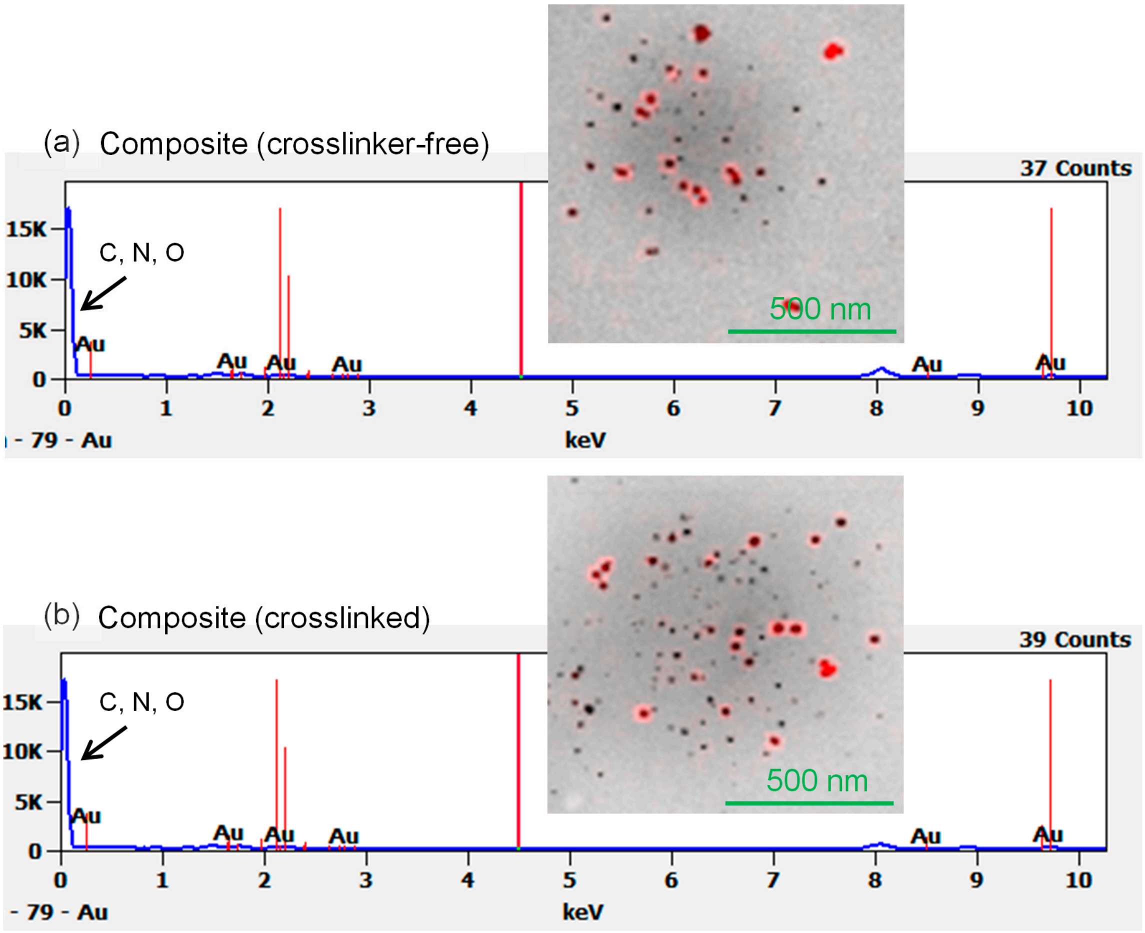

Figure S5. Energy dispersive x-ray maps and spectra for composite particles prepared with (a) crosslinker-free and (b) crosslinked PNIPAM particles.

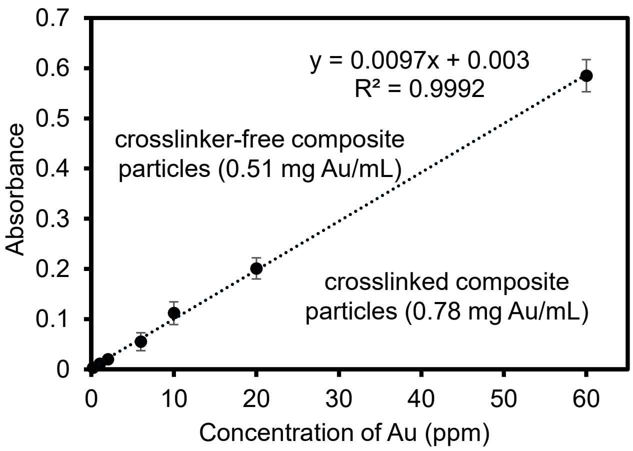

Figure S6. Calibration curve of Au atom standards by atomic absorption spectroscopy.

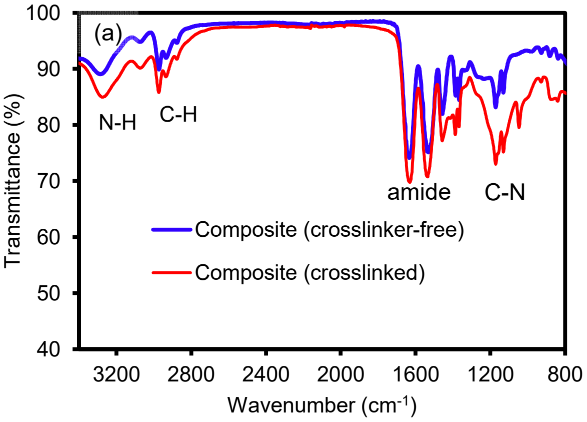

Obtained by a Spectrum 100 FT-IR Spectrometer (Perkin Elmer) equipped with an ATR sampling accessory under the following conditions (scan range: 4,000 to 650 cm^{-1}, resolution of 4 cm^{-1}, 64 scans).

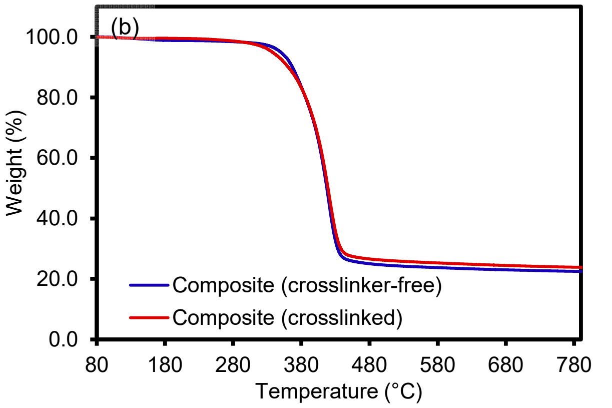

Obtained by a thermogravimetric analyzer (SDT Q600, TA Instrument) under the following conditions (10 min of pre-heating at 80 ℃, a ramping rate of 25 ℃ /min from 80 ℃ to 800 ℃, 50 mL/min N_2 gas flow).

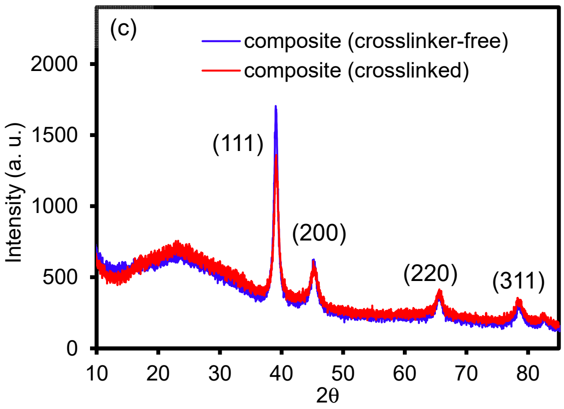

Obtained by a benchtop powder X-ray diffraction system with Cu K*α* radiation (MiniFlex 600, Rigaku Corp.) under the following conditions (scan rage: 5-85°, 0.02 steps, 5 degree/min)

Figure S7. Additional characterization of crosslinker-free and crosslinked composite particles by (a) Fourier transform infrared spectrometer, (b) thermogravimetric analyzer, and (c) powder x-ray diffractometer.

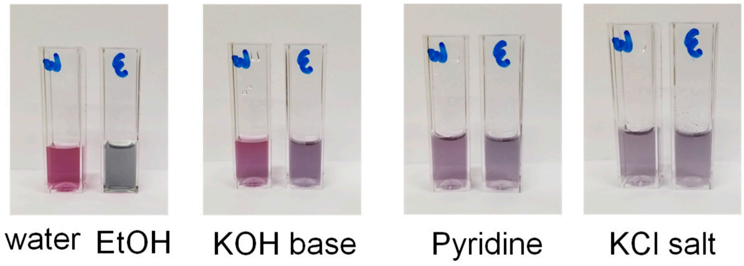

Figure S8. Stability of bare AuNPs in water and EtOH under various conditions.
